# Kefir

**Published:** 1884-08

**Authors:** 


### KEFIR.

WHILE during the last few years koumiss has been introduced into Western Europe, and even into America, a new drink prepared from cow's milk by a process of fermentation imperfectly understood is coming into use in Russia. This drink is kefir, and it has long formed the chief article of diet among the mountaineers in the neighborhood of Mount Elbruz and Kasbek, in the Caucasus. It forms a thick white fluid with a faintly acid flavor, said to resemble certain light wines. The mountaineers themselves call it "ghippo." The inhabitants of the plains near the Caucasus and the Russian settlers, who term it kefir, kifiir, or khiafar, make use of it, not for the table, but as a popular remedy for anæmia, struma, gastric catarrh, and chronic bronchitis.

According to the "Moscow Medical Gazette," where a contribution on the subject has recently appeared, Dr. Kern being the author, the preparation of kefir is very simple. The mountaineers make it by filling a bag made of goatskin with milk ; then a tenacious mass of the size of a walnut, of a material which they term "kefir seed," and the precise origin of which is unknown, is added to the milk. In a few hours the process of fermentation sets in actively. When prepared in wooden or glass vessels the kefir tastes better. After a lapse of twenty-four hours, a weak kefir is produced ; when the process is allowed to continue for three days the kefir becomes very strong. The source of the ferment is scrupulously concealed by the Caucasian mountaineers, who, with the humor of the English cook who once sold a secret for making "fundied cheese," the secret being that the cheese must be fundied after toasting and before the addition of pepper, cannot be persuaded to enlighten strangers to any greater extent than in supplying a small sample of the ferment, in the form of dry, dark-brown, earth-like masses, but steadfastly refusing to say whence they are obtained. One of these fragments dropped into milk begins rapidly to effervesce, turns milk-white, and assumes the form of a mulberry, then fermentation proceeds at once. If a piece, thus transformed, be dropped into another bowl of milk, it rapidly increases in size and also causes fermentation. Dr. Kern has carefully examined specimens of this "kefir seed," which consists chiefly of masses of zooglœa, holding together collections of a bacterium which he calls "Dispora caucasia." The yeast-fungus, "Saccharomyces cerevisiæ," is always found associated with this new germ. 'Kefir seed" retains its vitality after remaining for months in its dry condition. Dr. Kern has a great belief in the future of kefir, which has all the virtues of koumiss, and possesses one great advantage over the latter fluid in that it is just as good when prepared from cow's as from mare's milk.—[*British Medical Journal.*

